# Effects of the compound extracts of *Caprifoliaceae* and *Scutellaria baicalensis* Georgi on the intestinal microbiota and antioxidant function

**DOI:** 10.3389/fmicb.2023.1289490

**Published:** 2024-01-12

**Authors:** Shunfen Zhang, Huiyuan Lv, Xueying Cai, Shanlong Tang, Ruqing Zhong, Liang Chen, Hongfu Zhang

**Affiliations:** ^1^State Key Laboratory of Animal Nutrition and Feeding, Institute of Animal Science, Chinese Academy of Agricultural Sciences, Beijing, China; ^2^College of Animal Science and Technology, China Agricultural University, Beijing, China; ^3^Beijing Centre Biology Co., Ltd., Beijing, China; ^4^Hangzhou First People's Hospital, Hangzhou, China

**Keywords:** *Scutellaria baicalensis* Georgi, *Caprifoliaceae*, gut microbe, antioxidant, inflammatory

## Abstract

According to the Chinese encyclopedia “*Ben Cao Gang Mu*” (AD 1552–1578), *Caprifoliaceae* and *Scutellaria baicalensis* Georgi are used in traditional Chinese medicine to clear heat, detoxify, and treat wind-heat colds, upper respiratory tract infections, and pneumonia. However, the mechanism and the effects of the compound extracts of *Caprifoliaceae* and *Scutellaria baicalensis* Georgi on intestinal health remain unclear. From the perspective of intestinal microbes, this study assessed the antioxidant, anti-inflammatory, and intestinal protective properties of *Caprifoliaceae* and *Scutellaria baicalensis* Georgi. Mice received diets with or without *Caprifoliaceae* and *Scutellaria baicalensis* Georgi extractive (BCA) for 2 weeks in this study. The results showed that BCA increased body weight gain, feed intake, and catalase (CAT) content in the mice but reduced γ-glutamyl transpeptidase (γ-GT) content in the serum (*p* < 0.05). BCA improved the Sobs, Chao, and Ace indices, as well as the number of Campylobacterota, Patercibacteria, and Desulfobacterota in the colon microbiota, while it decreased the Firmicutes phylum (*p* < 0.05). At the genus level, BCA increased *Candidatus_Saccharimonas, Helicobacter, unclassified_f_Lachnospiraceae, Alistipes, norank_f_norank_o_Clostridia_vadinBB60_group, norank_f_Ruminococcaceae, unclassified_f_Ruminococcaceae*, etc. abundance (*p* < 0.05), but it significantly decreased *Lactobacillus* and *Lachnospiraceae_UCG_001* abundance (*p* < 0.05). Moreover, BCA improved the concentration of acetic acid, butyric acid, propionic acid, valeric acid, and isovaleric acid and diminished the concentration of isobutyric acid (*p* < 0.05). Correlation analysis shows that the changes in short-chain fatty acids and antioxidant and inflammatory indices in the serum were significantly correlated with the BCA-enriched microbiota. This study supplemented a database for the application of *Caprifoliaceae* and *Scutellaria baicalensis* Georgi in clinical and animal production.

## Graphical Abstract

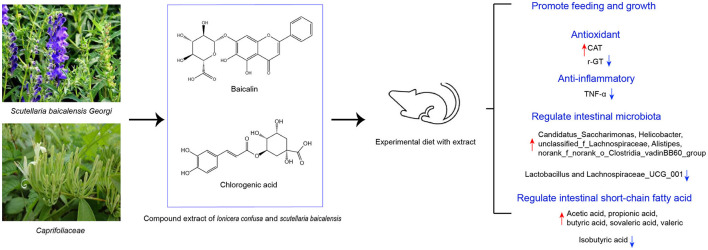

## 1 Introduction

Traditional Chinese medicine *Caprifoliaceae* and *Scutellaria baicalensis* Georgi, important natural active substances, have been widely used around the world (Zhao et al., 2019; Li et al., 2020). Pharmacological research has shown the antibacterial, anti-inflammatory, antiviral, antioxidant, and liver protection activities of *Caprifoliaceae* (Shi et al., 2016). One extractive for *Caprifoliaceae* is chlorogenic acid, a phenylpropanoid compound that has extensive biological activities including antibacterial, antiviral, and antioxidant (Miao and Xiang, 2020). *Scutellaria baicalensis* Georgi is effective in the treatment of respiratory infections, gastroenteritis, and diarrhea (Zhao et al., 2016). The main extractive of *Scutellaria baicalensis* Georgi is baicalin, a class of flavonoids that has been reported to have anti-inflammatory and immune-regulatory activities (Wang et al., 2022). The mixture of baicalin and chlorogenic acid could synergistically deliver a stronger antioxidant and anti-inflammatory effect.

The intestinal microbiota is critical for regulating host nutrition, metabolism, intestinal microbiota balance, and immune function (Maynard et al., 2012). *In vivo*, the intestinal microbiota widely participates in the metabolism of traditional Chinese medicine. After oral administration, baicalin or other traditional Chinese medicines are degraded by the intestinal microbiota and produce aglycones that have pharmacological effects (Huang et al., 2019). The intestinal microbiota takes part in herb metabolism by producing various enzymes and then changing the trend of medicines' action (Huang et al., 2018). Therefore, the research on the effects of Chinese herbal medicines on the intestinal microbiota to understand their mechanism of treating disease *in vivo* is a visionary effort.

Baicalin and chlorogenic acid are hardly absorbed in the small intestine and are stuck in the intestine for a long period of time. As a result, they are mostly broken down by microbiota and susceptible to the influence of microbiota. In this study, 16S rRNA gene sequencing analysis was used to investigate the effects of the compound extracts of *Caprifoliaceae* and *Scutellaria baicalensis* Georgi on the intestinal microbiota and its metabolites. The purpose of this study was to provide a database for the application of BCA in clinical and animal production.

## 2 Materials and methods

### 2.1 Characteristics of the compound extract of *Caprifoliaceae* and *Scutellaria baicalensis* Georgi

The compound extract of *Caprifoliaceae* and *Scutellaria baicalensis* Georgi was supplied by Beijing Center Biology Co., Ltd, of which the core components are baicalin and chlorogenic acid, and the ratio was 1:10. The purity of baicalin and chlorogenic acid was 85% and 65%, respectively. The plant name has been checked with “World Flora Online” (www.worldfloraonline.org).

### 2.2 Mice and experiment design

In total, 24 Institute of Cancer Research (ICR) mice (21-day-old) were purchased from the School of Medicine, Peking University (Beijing, China) and randomly assigned into a control group (CON) and a compound extract of *Caprifoliaceae* and *Scutellaria baicalensis* Georgi group (BCA). The mice in the CON group were given regular food, while the mice in the BCA group were given food with 500 mg/kg of BCA for 2 weeks. The mice were allowed to eat and drink freely during the experiment.

Blood was collected by removing the eyeball and then centrifuged for 10 min at 3,000 rpm under 4°C to separate the serum. In post-trial, the mice were euthanized using the cervical dislocation method, and the jejunum, colon, and colonic chyme were excised for processing. The experimental protocol was reviewed and approved by the Institutional Animal Care and Use Committee of the Institute of Animal Science at the Chinese Academy of Agricultural Sciences (IAS2020-85).

### 2.3 Intestinal morphology

The jejunum and colon tissue samples were prepared on tissue slides according to the procedure reported earlier (Tang et al., 2022). The tissues were embedded in paraffin blocks, cut into 4-μm slices, and stained with hematoxylin and eosin (H&E). Then, the slices were dried and sealed for follow-up observation and analysis.

### 2.4 Serum antioxidant capacity and inflammatory cytokines analysis

The activities of γ-glutamyl transpeptidase (γ-GT, cat. no. C017-2-1), catalase (CAT, cat. no. A007-2-1), malondialdehyde (MDA, cat. no. A003-1-2), total antioxidant capacity (T-AOC, cat. no. A015-2-1), and diamine oxidase (DAO, cat. no. A088-1-1) were performed via appropriate assay kits (Nanjing Jiancheng Bioengineering Institute, Nanjing, China) using the builder's standard method. In addition, interleukin-1β (IL-1β, cat. no. H002-1-2), interleukin-6 (IL-6, cat. no. H007-1-1), and tumor necrosis factor-α (TNF-α, cat. no. H052-1-1) levels in the serum were tested via the enzyme-linked immunosorbent assay (ELISA) (Nanjing Jiancheng Bioengineering Institute, Nanjing, China) according to the manufacturer's instructions.

### 2.5 16s rRNA gene sequencing analysis

Microbial DNA in colonic chyme was obtained using the E.Z.N.A.^®^ soil DNA kit (Omega Bio-Tek, Norcross, GA, United States), according to the builder's standard method. The V3-V4 hypervariable regions of the microbial 16S rRNA gene were amplified with PCR using primer pairs 338F (5′-ACTCCTACGGGAGGCAGCAG-3′) and 806R (5′-GGACTACHVGGGTWTCTAAT-3′) via the ABI Gene Amp^®^ 9700 PCR thermocycler (ABI, CA, United States). Metagenomic sequencing was executed according to the Illumina platform by Miseq PE300. Raw reads were deposited into the NCBI Sequence Read Archive database (SRA: PRJNA765910). Raw sequences were treated and filtered into chimeric sequences by the Majorbio I-Sanger Cloud Platform (www.i-sanger.com, Majorbio, Shanghai, China). The Majorbio I-Sanger Cloud Platform (www.i-sanger.com, Majorbio, Shanghai, China) was used to perform unweighted principal coordinate analysis (PCoA), beta-diversity analysis, and alpha-diversity analysis using default values.

### 2.6 Short-chain fatty acid analysis

Colon chyme samples were used to test short-chain fatty acids (SCFAs) concentrations using a gas chromatography-mass spectrometer (GC-MS), as described by Wan et al. (2022). Colon chyme samples of 0.1 g were weighed, suspended in 1 ml of ddH_2_O, homogenized, and centrifuged (10,000 rpm, 10 min, 4°C). The supernatant was obtained, and 25% of metaphosphoric acid was added. Then, the supernatant was filtered and analyzed using an Agilent 6890 gas chromatography (Agilent Technologies, Inc., Palo Alto, CA, United States) system.

### 2.7 Statistical analysis

Quantitative data are expressed as the mean ± standard deviation (SD). The SAS 9.4 software (SAS 9.4, Institute, Cary, NC, United States) was used for all the analyses. Comparisons of means between the BCA and CON groups were performed using an unpaired *t*-test. Significance was established at *p* ≤ 0.05.

## 3 Results

### 3.1 Growth performance and intestinal morphology

**Figure 1:**
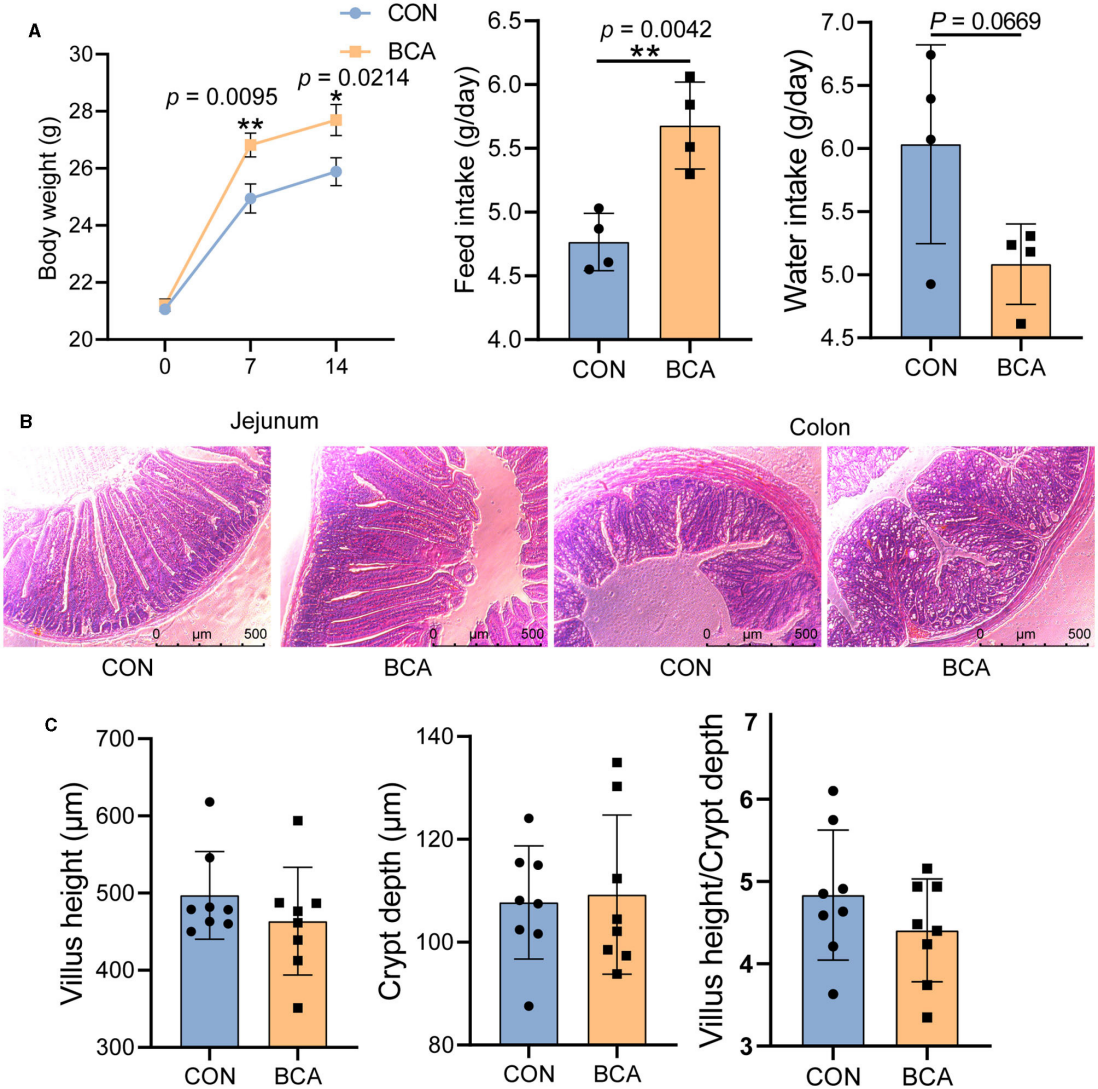
Growth performance and intestinal morphology. **(A)** Body weight (*n* = 12), feed intake, and water intake; **(B)** staining profiles by H&E of the jejunum and colon (scale bars: 500 μm); and **(C)** villus height, crypt depth, and villus height/crypt depth of jejunum, *n* = 8. Data are expressed as mean with SD. The intergroup difference test was performed using an unpaired t-test. ^*^means *p* < 0.05; ^**^means *p* < 0.01.

BCA significantly increased body weight gain and feed intake of the mice (Figure 1A, *p* < 0.05), but tended to reduce water intake (*p* = 0.0669). Intestinal morphology was intact both in the control and BCA groups, and villi and crypts were neatly arranged (Figures 1B, C).

### 3.2 Serum biochemical parameters and inflammatory cytokines

**Figure 2:**
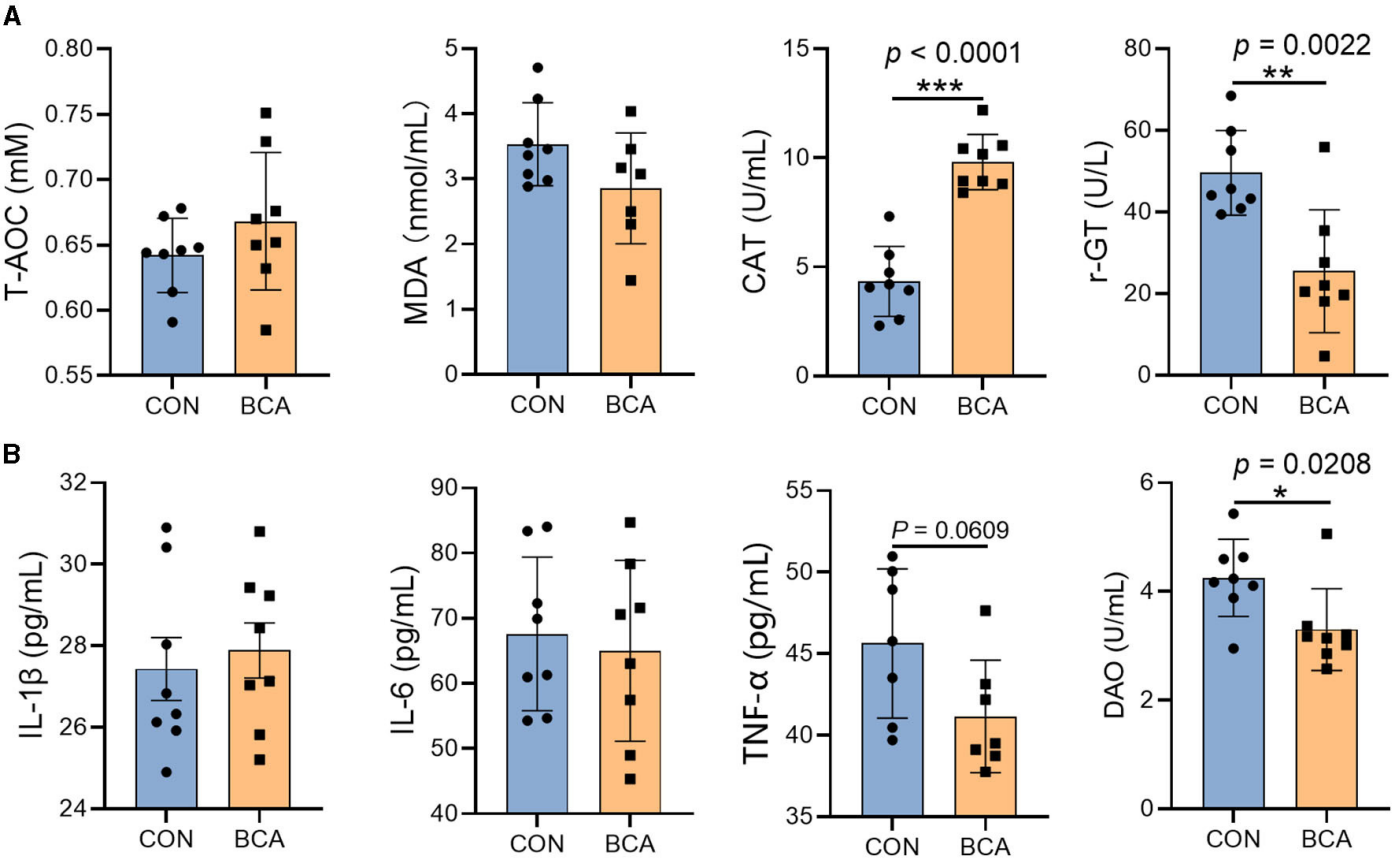
Serum biochemical parameters and inflammatory cytokines. **(A)** Antioxidant enzymes (T-AOC, MDA, CAT, and γ-GT) and **(B)** inflammatory cytokines (IL-1β, IL-6, and TNF-α) and DAO in the serum. Data are expressed as mean with SD. The intergroup difference test was performed using an unpaired *t*-test (*n* = 8). *means *p* < 0.05; **means *p* < 0.01; ***means *p* < 0.001.

BCA significantly reduced the γ-GT content in the serum (Figure 2A, *p* < 0.05) but increased CAT content (*p* < 0.05). In addition, BCA significantly reduced DAO content (*p* < 0.05) and tended to reduce the TNF-α content in the serum (Figure 2B, *p* = 0.0609).

### 3.3 Colonic microbiota

**Figure 3:**
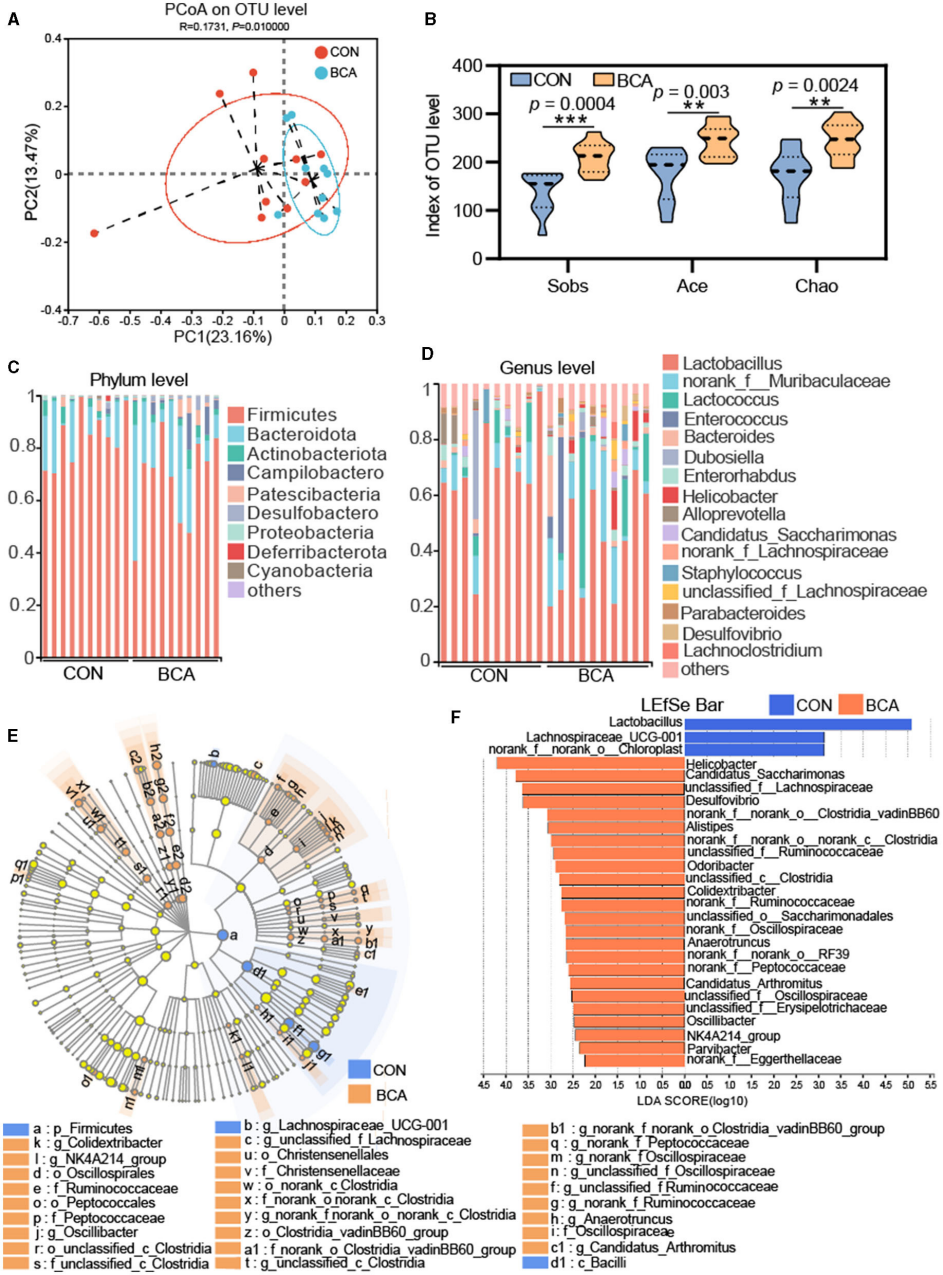
Microbiota analysis in colon chyme (*n* = 10). **(A)** PCoA analysis based on unweighted UniFrac. **(B)** Alpha-diversity (Sobs, Chao, and Ace indices) of the microbiota. Data are expressed as mean with SD. The intergroup difference test was performed using an unpaired *t*-test. **means *p* < 0.01; ***means *p* < 0.001. **(C)** Relative abundance of the microbiome at phylum level and **(D)** genus level. Less than 1% abundance of phyla or genera was merged into others. **(E)** LEfSe analysis at the genus level and the threshold of the LDA score was 2.0. Blue represents the genus enriched by CON, and orange represents the genus enriched by BCA. **(F)** Lefse bar in genus levels in CON vs. BCA.

Figure 3 shows the effect of BCA on the diversity of the colonic microbiota. A PCoA analysis revealed that there was a clear cluster of the microbial community at the OTU level between the BCA and CON groups (Figure 3A). For alpha diversity, BCA significantly improved the sobs, ace, and chao indices (*p* < 0.01, Figure 3B).

Microbial composition is shown in Figures 3C, D. Firmicutes, Actinobacteriota, Patescibacteria, and Bacteroidota were abundant in both CON and BCA groups. Campylobactero and Desulfobactero were important components for the BCA group but not for the CON group. At the genus level, the top six abundance genera in the CON group were *Lactobacillus, norank_f__Muribaculaceae, Lactococcus, Dubosiella, Enterorhabdus*, and *Alloprevotella*. The top six abundance genera in the BCA group were *Lactobacillus, norank_f__Muribaculaceae, Lactococcus, Enterococcus, Bacteroides*, and *Helicobacter*. Linear discriminate analysis effect size (LEfSe) was used to identify the specific taxa from phylum to genus level, and the threshold of the linear discriminant analysis (LDA) score was 2.0. Firmicutes and Bacilli were identified in the CON group. A total of 6, 7, or 15 microbiotas were identified in the BCA group at the order, family, and genus levels, respectively. The LEfSe bar shows the identified genus in the CON and BCA groups. The top five abundant genera of identified taxa in the BCA group were *Helicobacter, Candidatus_Saccharimonas, unclassified_f_Lachnospiraceae, Desulfovibrio*, and *norank_f_norank_o_Clostridia_vadinBB60_group*.

**Figure 4:**
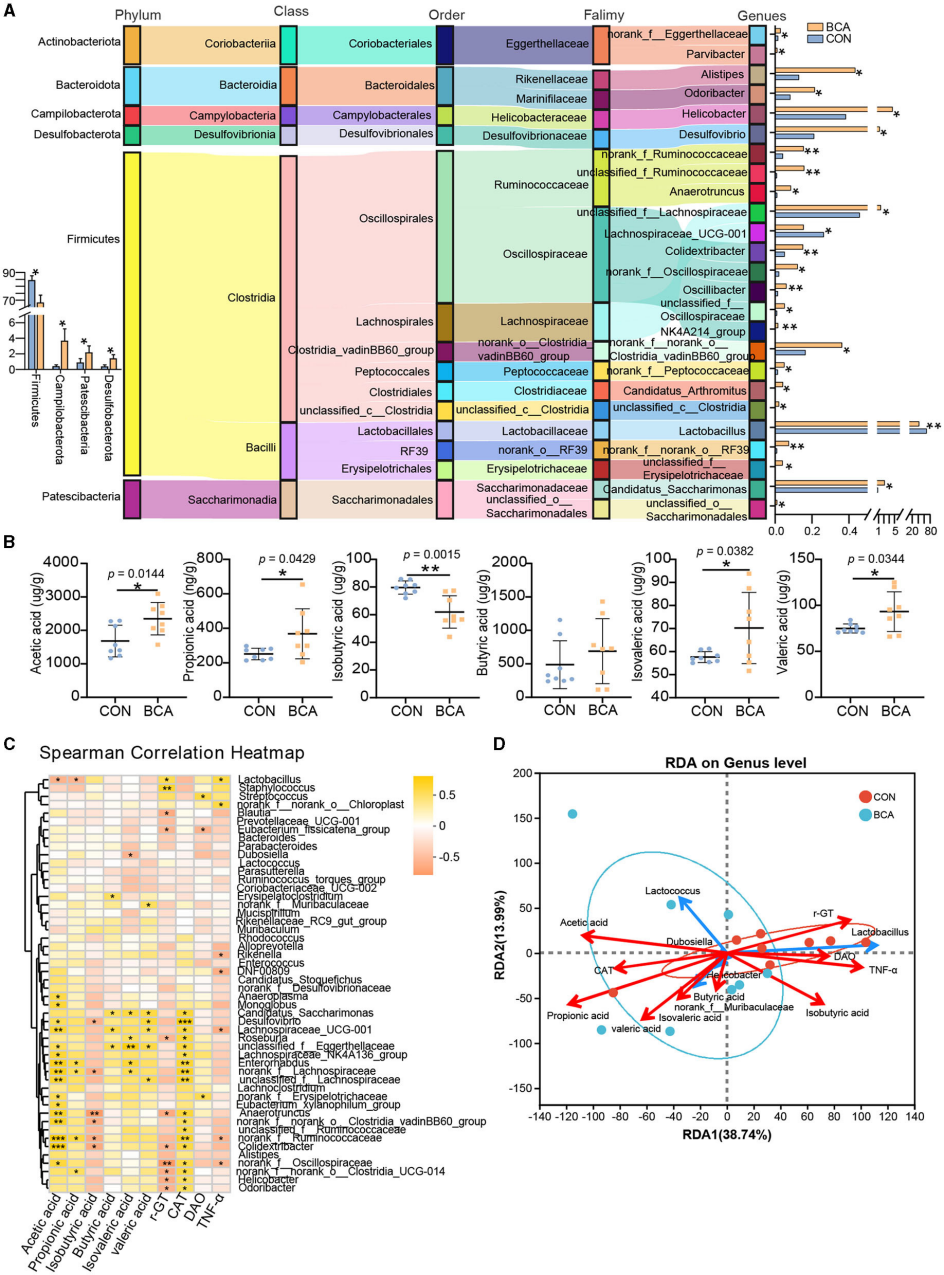
Differential genera and short-chain fatty acid analysis. **(A)** Taxonomy of differentially abundant genera in the BCA vs. CON group from phylum to genus level. **(B)** Short-chain fatty acid concentration of colon chyme (*n* = 8). Data are expressed as mean with SD. The intergroup difference test was performed using an unpaired *t*-test. **(C)** Correlation analysis between SCFAs or serum index and the top 50% genera was performed using Spearman. **(D)** Redundancy analysis (RDA) of the colonic microbiota composition at the genus level relative to colonic SCFAs and serum index. The top five microbes are shown. *means *p* < 0.05, **means *p* < 0.01, ***means *p* < 0.001.

As shown in Figure 4A, 92% of the identified genera were improved in the BCA group (*p* < 0.05), among which the top 10 abundant genera were *Candidatus_Saccharimonas, Helicobacter, Desulfovibrio, unclassified_f_Lachnospiraceae, Alistipes, norank_f_norank_o_Clostridia_vadinBB60_group, Odoribacter, norank_f_Ruminococcaceae, unclassified_f_Ruminococcaceae*, and *Colidexinbater*. In contrast, *Lactobacillus* and *Lachnospiraceae_UCG_001* were significantly reduced in the BCA group (*p* < 0.05). More than 65% of altered microbiotas belong to the Firmicutes phylum, and 82% of them belong to the Clostridia class. At the phylum level, BCA significantly reduced Firmicutes abundance but increased Campylobacterota, Patercibacteria, and Desulfobacterota abundance.

### 3.4 Short-chain fatty acid production

SCFAs quantification in colon chyme from CON and BCA groups was measured and depicted in Figure 4B. BCA treatment did improve the concentration of acetic acid, butyric acid, propionic acid, valeric acid, and isovaleric acid and diminish the concentration of isobutyric acid (*p* < 0.05).

### 3.5 Correlation analysis

The correlation heatmap revealed the correlation conducted using Spearman correlation analysis between SCFAs or serum index and microbiotas in Figure 4C. The acetic acid level and CAT content in the serum had a positive correlation with the abundance of *Lachnospiraceae_UCG_001, Desulfovibrio, Colidexinbater, norank_f_Ruminococcaceae, unclassified_f_Eggerthellaceae, Lachnospiraceae_NK4A136_group, unclassified_f_Lachnospiraceae, norank_f_Lachnospiraceae, Enterorhabdus, Monoglobus, Anaerotruncus, norank_f_norank_o_Clostridia_vadinBB60_group, Eubacterium_xylanophilum_group, norank_f_Erysipelotrichaceae, norank_Oscillospiraceae*, and *Anaeropiasma*, but a negative correlation with the abundance of *Lactobacillus*. The concentration of propionic acid had a positive correlation with the abundance of *norank_f_Ruminococcaceae, norank_f_Lachnospiraceae, Enterorhabdus*, and *norank_f_norank_o_Clostridia_UCG_014*, but a negative correlation with the abundance of *Lactobacillus*. The concentration of isobutyric acid was a negative correlation with the abundance of *Desulfovibrio, Colidexinbater, norank_f_Ruminococcaceae, norank_f_Lachnospiraceae, Anaeropiasma*, and *norank_f_ norank_o_Clostridia_vadinBB60_group*. The concentration of butyric acid, isovaleric acid, and valeric acid had a positive correlation with *Lachnospiraceae_UCG_001, Desulfovibrio, Candidatus_Saccharimonas, unclassified_f_Eggerthellaceae*, etc. γ-GT content in the serum had a negative correlation with *norank_Oscillospiraceae, Alistipes, Helicobacter*, etc. To further explore the association among bacteria, SCFAs, or serum index, RDA analysis was completed. The top five bacteria are shown in Figure 4D. The results showed that the BCA group was separated from those in the control group. Genera (*Lactococcus, Helicobacter, Dubosiella*, and *norank_f_Muribaculaceae*) enriched in the BCA group were positively correlated with acetic acid, propionic acid, butyric acid, isovaleric acid, valeric acid, and CAT. In contrast, genera (*Lactobacillus*) enriched in the CON group were positively correlated with isobutyric acid, γ-GT, DAO, and TNF-α.

## 4 Discussion

*Caprifoliaceae* and *Scutellaria baicalensis* Georgi have high medicinal value and are widely used in traditional Chinese medicine. Chlorogenic acid, the main component of *Caprifoliaceae*, and baicalin, the main component of *Scutellaria baicalensis* Georgi, have also received extensive attention. In the field of animal production, baicalin has been reported to improve the performance of pigs and chickens and improve intestinal diseases (Yin et al., 2021). Chlorogenic acid has been reported to have antibacterial, antiviral, and microbiota-regulation effects (Kim et al., 2020). In this study, BCA promoted the body weight of the mice. This may be related to the improvement of the feed intake, intestinal health, and immunity of mice after BCA treatment. In a previous study, we found that baicalin regulates appetite-related genes and promotes feed intake in mice (Zhang et al., 2023). Chlorogenic acid supplementation upregulates anti-inflammatory cytokines and antioxidant enzymes and improves DSS-induced colitis through the Nrf2 signaling pathway (Wan et al., 2021). DAO levels in the serum generally remain low, which increased when the intestinal barrier was compromised (Lu et al., 2022). In this study, lower DAO levels in the serum after BCA treatment may indicate enhanced epithelial integrity of the intestinal barrier (Yang et al., 2019). In addition, the intestinal microbiota has been extensively reported to be closely related to host intestinal digestion and absorption (Khudhair et al., 2020). BCA supplementation in this study increased the colonization of Firmicutes, especially Clostridia and other probiotics. BCA-enriched bacteria Ruminococcaceae have been reported to reduce intestinal permeability and are involved in food digestion and carbohydrate metabolism (Xi et al., 2021). *Lactococcus*, enriched in the BCA group, has been widely reported to promote animal growth and secrete antimicrobial peptides and organic acids to inhibit the growth of pathogenic bacteria and maintain intestinal homeostasis (Feng et al., 2019; Kakade et al., 2022; Werum et al., 2022). *Lactococcus* also regulated host immunity by secreting exopolysaccharides and was considered a probiotic due to its ability to inhibit the growth of pathogenic bacteria (Sun et al., 2020; Pan et al., 2022).

Baicalin and chlorogenic acid play an antioxidant role in the phenolic hydroxyl structure, which effectively removes hydroxyl radicals and superoxide anions generated in the free radical reaction by reacting easily with free radicals (Xia et al., 2021). In the present research, BCA significantly improved the antioxidant function and reduced inflammation in mice, including reducing the content of γ-GT and TNF-α and improving the content of CAT in the serum. In addition to the special structure and direct effects of chlorogenic acid and baicalin, the anti-inflammatory functions of BCA may also be related to the intestinal microbiota, which has been widely reported (Negatu et al., 2020; Shen et al., 2022). The correlation and RDA analyses showed that antioxidant and inflammatory indicators were significantly correlated with BCA-enriched intestinal microbiota in this study. Some of these microbiotas may be associated with anti-inflammatory and antioxidant functions. *Lachnospiraceae_NK4A136_group* shows anti-inflammatory properties, promoting the repair of the intestinal mucosa and relieving colitis (Zhong et al., 2021). *norank_f_Muribaculaceae* could improve intestinal mucositis in mice (Wang et al., 2016). Above all, BCA supplementation improved antioxidant and anti-inflammatory function in mice. We speculate that the antioxidant and anti-inflammatory functions of BCA are partly attributed to the functional components baicalin and chlorogenic acid and whether the synergistic effects of the two are more worthy of further investigation.

SCFAs have been widely shown to maintain intestinal balance and actively participate in immune regulation, cell proliferation, and host metabolism (Akhtar et al., 2022; Li et al., 2022). Acetic acid could be taken up and utilized by many tissues, which was the main way for tissues to use carbohydrates that cannot be absorbed by the small intestine. Butyric acid, the most important energy source for the colon and cecum, was absorbed by the epithelial cells. In this study, BCA treatment significantly increased the contents of acetic acid, isovaleric acid, propionic acid, and valeric acid in the colonic chyme of mice. Higher concentrations of SCFAs inhibit pathogenic bacteria growth by reducing intestinal pH (He et al., 2019). After BCA intervention, SCFA-producing bacteria were abundant in the colon, which could promote microbial fermentation to produce more SCFAs and other metabolites. These metabolites could participate in intestinal development, immune regulation, digestion, and absorption by the body. This was confirmed by the correlation analysis between SCFA and the intestinal microbiota. Interestingly, most of the microbes associated with SCFAs and CAT belong to Firmicutes, especially Clostridia. *Alistipes* could produce indoles, acetic acid, and propionic acid and promote broilers' growth performance, which was considered to be potentially beneficial (Parker et al., 2020). *norank_Oscillospiraceae*, a butyric acid producer, could use gluconates (Leth et al., 2022). *unclassified_f_Ruminococcaceae* and *norank_f__Ruminococcaceae* belong to the Ruminococcaceae family and are the cause of SCFAs from carbohydrate or oligosaccharide degradation (Cheng et al., 2018). In addition, Ruminococcaceae was also a potential probiotic that could improve immunity and the intestinal environment (Gu et al., 2022).

In summary, BCA regulates the gut microbiota (increases Firmicutes, especially Clostridia), improves antioxidant function, and promotes feed intake and growth performance in mice. These results reveal the potential value of BCA in intestine protection and antioxidant function. However, whether the antioxidant function of BCA is achieved by gut microbes and the efficacy of these microbes in antioxidant and intestine protection remains to be further explored.

## Data availability statement

The datasets presented in this study can be found in online repositories. The names of the repository/repositories and accession number(s) can be found in the article/Supplementary material.

## Ethics statement

The animal study was approved by Institutional Animal Care and Use Committee of the Institute of Animal Science at the Chinese Academy of Agricultural Sciences. The study was conducted in accordance with the local legislation and institutional requirements.

## Author contributions

SZ: Conceptualization, Data curation, Formal analysis, Investigation, Methodology, Project administration, Software, Validation, Visualization, Writing—original draft, Writing—review & editing. HL: Data curation, Formal analysis, Funding acquisition, Investigation, Methodology, Supervision, Writing—review & editing. XC: Conceptualization, Data curation, Formal analysis, Funding acquisition, Investigation, Software, Writing—review & editing. ST: Conceptualization, Data curation, Investigation, Writing—review & editing. RZ: Conceptualization, Data curation, Investigation, Methodology, Writing—review & editing. LC: Data curation, Formal analysis, Funding acquisition, Methodology, Project administration, Resources, Writing—review & editing. HZ: Funding acquisition, Project administration, Resources, Supervision, Validation, Visualization, Writing—review & editing.
