# Supplementary material for: Effects of the compound extracts of Caprifoliaceae and Scutellaria baicalensis Georgi on the intestinal microbiota and antioxidant function
Source: Front Microbiol. 2024 Jan 12;14:1289490. doi: 10.3389/fmicb.2023.1289490 (PMC10822692; doi:10.3389/fmicb.2023.1289490)
Supplement: Supplementary file 2 [file Table_2.DOCX]

**Statistical programs**

**Body weight day0**

**data** d;

input group $ d @@;

datalines;

CON 22.09

CON 20.58

CON 21.06

CON 21.17

CON 21.27

CON 20.06

CON 21.6

CON 20.8

CON 20.38

CON 21.64

CON 20.48

CON 21.6

BCA 20.53

BCA 20.86

BCA 21.92

BCA 21.53

BCA 22.73

BCA 20.64

BCA 21.98

BCA 21.25

BCA 20.24

BCA 20.61

BCA 21.27

BCA 21.01

;

**proc** **ttest** data=d;

class group;

var d;

**run**;

**Results**

The TTEST Procedure

Variable: d

| group | N | Mean | Std Dev | Std Err | Minimum | Maximum |  |
| --- | --- | --- | --- | --- | --- | --- | --- |
| BCA | 12 | 21.2142 | 0.7240 | 0.2090 | 20.2400 | 22.7300 |  |
| CON | 12 | 21.0608 | 0.6130 | 0.1770 | 20.0600 | 22.0900 |  |
| Diff (1-2) |  | 0.1533 | 0.6708 | 0.2738 |  |  |  |

| group | Method | Mean | 95% CL Mean | | Std Dev | 95% CL Std Dev | |
| --- | --- | --- | --- | --- | --- | --- | --- |
| BCA |  | 21.2142 | 20.7542 | 21.6742 | 0.7240 | 0.5129 | 1.2292 |
| CON |  | 21.0608 | 20.6714 | 21.4503 | 0.6130 | 0.4342 | 1.0408 |
| Diff (1-2) | Pooled | 0.1533 | -0.4146 | 0.7213 | 0.6708 | 0.5188 | 0.9494 |
| Diff (1-2) | Satterthwaite | 0.1533 | -0.4155 | 0.7222 |  |  |  |

| Method | Variances | DF | T Value | Pr > \|t\| |
| --- | --- | --- | --- | --- |
| Pooled | Equal | 22 | 0.56 | 0.5812 |
| Satterthwaite | Unequal | 21.418 | 0.56 | 0.5813 |

| Equality of Variances | | | | |
| --- | --- | --- | --- | --- |
| Method | Num DF | Den DF | F Value | Pr > F |
| Folded F | 11 | 11 | 1.39 | 0.5904 |

**Body weight day 7**

**data** d;

input group $ d @@;

datalines;

CON 24.61

CON 22.74

CON 26.45

CON 25.8

CON 26.66

CON 23.77

CON 25.09

CON 21.7

CON 23.36

CON 25.9

CON 27.69

CON 25.6

BCA 29.36

BCA 27.09

BCA23.45

BCA 26.84

BCA 27.61

BCA 27.98

BCA 26.69

BCA 26.7

BCA 26.98

BCA 25.68

BCA 27.74

BCA 25.74

;

**proc** **ttest** data=d;

class group;

var d;

**run**;

**Results**

The TTEST Procedure

Variable: d

| group | N | Mean | Std Dev | Std Err | Minimum | Maximum |
| --- | --- | --- | --- | --- | --- | --- |
| BCA | 12 | 26.8217 | 1.4529 | 0.4194 | 23.4500 | 29.3600 |
| CON | 12 | 24.9475 | 1.7641 | 0.5092 | 21.7000 | 27.6900 |
| Diff (1-2) |  | 1.8742 | 1.6160 | 0.6597 |  |  |

| group | Method | Mean | 95% CL Mean | | Std Dev | 95%CLStdDev | |
| --- | --- | --- | --- | --- | --- | --- | --- |
| BCA |  | 26.8217 | 25.8985 | 27.7448 | 1.4529 | 1.0292 | 2.4669 |
| CON |  | 24.9475 | 23.8267 | 26.0683 | 1.7641 | 1.2497 | 2.9952 |
| Diff (1-2) | Pooled | 1.8742 | 0.5060 | 3.2424 | 1.6160 | 1.2498 | 2.2872 |
| Diff (1-2) | Satterthwaite | 1.8742 | 0.5031 | 3.2453 |  |  |  |

| Method | Variances | DF | T Value | Pr > \|t\| |
| --- | --- | --- | --- | --- |
| Pooled | Equal | 22 | 2.84 | 0.0095 |
| Satterthwaite | Unequal | 21.221 | 2.84 | 0.0097 |

| Equality of Variances | | | | |
| --- | --- | --- | --- | --- |
| Method | Num DF | Den DF | F Value | Pr > F |
| Folded F | 11 | 11 | 1.47 | 0.5305 |

**Body weight day14**

**data** d;

input group $ d @@;

datalines;

CON 25.28

CON 24.88

CON 26.17

CON 26.34

CON 28.76

CON 25.23

CON 25.87

CON 25.62

CON 23.66

CON 26.32

CON 29.05

CON 23.44

BCA 31.05

BCA 24.4

BCA 27.06

BCA 27.17

BCA 29.29

BCA 29.26

BCA 25.97

BCA 27.2

BCA 28.51

BCA 25.38

BCA 28.04

BCA 29.02

;

**proc** **ttest** data=d;

class group;

var d;

**run**;

**Results**

The TTEST Procedure

Variable: d

| group | N | Mean | Std Dev | Std Err | Minimum | Maximum |
| --- | --- | --- | --- | --- | --- | --- |
| BCA | 12 | 27.6958 | 1.8806 | 0.5429 | 24.4000 | 31.0500 |
| CON | 12 | 25.8850 | 1.6940 | 0.4890 | 23.4400 | 29.0500 |
| Diff (1-2) |  | 1.8108 | 1.7897 | 0.7307 |  |  |

| group | Method | Mean | 95% CL Mean | | Std Dev | 95% CL Std Dev | |
| --- | --- | --- | --- | --- | --- | --- | --- |
| BCA |  | 27.6958 | 26.5009 | 28.8907 | 1.8806 | 1.3322 | 3.1931 |
| CON |  | 25.8850 | 24.8087 | 26.9613 | 1.6940 | 1.2000 | 2.8761 |
| Diff (1-2) | Pooled | 1.8108 | 0.2956 | 3.3261 | 1.7897 | 1.3842 | 2.5331 |
| Diff (1-2) | Satterthwaite | 1.8108 | 0.2946 | 3.3271 |  |  |  |

| Method | Variances | DF | T Value | Pr > \|t\| |
| --- | --- | --- | --- | --- |
| Pooled | Equal | 22 | 2.48 | 0.0214 |
| Satterthwaite | Unequal | 21.764 | 2.48 | 0.0214 |

| Equality of Variances | | | | |
| --- | --- | --- | --- | --- |
| Method | Num DF | Den DF | F Value | Pr > F |
| Folded F | 11 | 11 | 1.23 | 0.7349 |

**Feed intake**

**data** d;

input group $ d @@;

datalines;

CON 5.030962

CON 4.869038

CON 4.607308

CON 4.551538

BCA 5.295962

BCA 5.512115

BCA 5.843654

BCA 6.062692

;

**proc** **ttest** data=d;

class group;

var d;

**run**;

**Results**

The TTEST Procedure

Variable: d

| group | N | Mean | StdDev | StdErr | Minimum | Maximum |
| --- | --- | --- | --- | --- | --- | --- |
| BCA | 4 | 5.6786 | 0.3410 | 0.1705 | 5.2960 | 6.0627 |
| CON | 4 | 4.7647 | 0.2251 | 0.1125 | 4.5515 | 5.0310 |
| Diff (1-2) |  | 0.9139 | 0.2889 | 0.2043 |  |  |

| group | Method | Mean | 95% CL Mean | | Std Dev | 95% CL Std Dev | |
| --- | --- | --- | --- | --- | --- | --- | --- |
| BCA |  | 5.6786 | 5.1360 | 6.2213 | 0.3410 | 0.1932 | 1.2715 |
| CON |  | 4.7647 | 4.4065 | 5.1229 | 0.2251 | 0.1275 | 0.8392 |
| Diff (1-2) | Pooled | 0.9139 | 0.4140 | 1.4138 | 0.2889 | 0.1862 | 0.6362 |
| Diff (1-2) | Satterthwaite | 0.9139 | 0.3946 | 1.4332 |  |  |  |

| Method | Variances | DF | T Value | Pr > \|t\| |
| --- | --- | --- | --- | --- |
| Pooled | Equal | 6 | 4.47 | 0.0042 |
| Satterthwaite | Unequal | 5.1969 | 4.47 | 0.0060 |

| Equality of Variances | | | | |
| --- | --- | --- | --- | --- |
| Method | Num DF | Den DF | F Value | Pr > F |
| Folded F | 3 | 3 | 2.30 | 0.5127 |

**Water intake**

**data** d;

input group $ d @@;

datalines;

CON 6.742885

CON 6.395577

CON 4.925962

CON 6.072308

BCA 5.235577

BCA 5.182308

BCA 4.6125

BCA 5.307885

;

**proc** **ttest** data=d;

class group;

var d;

**run**;

**Results**

The TTEST Procedure

Variable: d

| group | N | Mean | Std Dev | Std Err | Minimum | Maximum |
| --- | --- | --- | --- | --- | --- | --- |
| BCA | 4 | 5.0846 | 0.3189 | 0.1594 | 4.6125 | 5.3079 |
| CON | 4 | 6.0342 | 0.7879 | 0.3940 | 4.9260 | 6.7429 |
| Diff (1-2) |  | -0.9496 | 0.6010 | 0.4250 |  |  |

| group | Method | Mean | 95% CL Mean | | Std Dev | 95% CL Std Dev | |
| --- | --- | --- | --- | --- | --- | --- | --- |
| BCA |  | 5.0846 | 4.5771 | 5.5920 | 0.3189 | 0.1806 | 1.1890 |
| CON |  | 6.0342 | 4.7804 | 7.2879 | 0.7879 | 0.4464 | 2.9378 |
| Diff (1-2) | Pooled | -0.9496 | -1.9896 | 0.0903 | 0.6010 | 0.3873 | 1.3235 |
| Diff (1-2) | Satterthwaite | -0.9496 | -2.1347 | 0.2354 |  |  |  |

| Method | Variances | DF | T Value | Pr > \|t\| |
| --- | --- | --- | --- | --- |
| Pooled | Equal | 6 | -2.23 | 0.0669 |
| Satterthwaite | Unequal | 3.9571 | -2.23 | 0.0899 |

| Equality of Variances | | | | |
| --- | --- | --- | --- | --- |
| Method | Num DF | Den DF | F Value | Pr > F |
| Folded F | 3 | 3 | 6.10 | 0.1715 |

**Villus height**

data d;

input group $ d @@;

datalines;

CON 478.8785

CON 478.388

CON 545.725

CON 450.092

CON 463.025

CON 481.84

CON 460.1011

CON 618.192

BCA 412.506

BCA 351.0757

BCA 487.5913

BCA 476.372

BCA 439.265

BCA 593.88

BCA 461.5483

BCA 486.8811

;

proc ttest data=d;

class group;

var d;

run;

**Results**

The TTEST Procedure

Variable: d

| group | N | Mean | StdDev | StdErr | Minimum | Maximum |
| --- | --- | --- | --- | --- | --- | --- |
| BCA | 8 | 463.6 | 69.8184 | 24.6845 | 351.1 | 593.9 |
| CON | 8 | 497.0 | 56.9240 | 20.1257 | 450.1 | 618.2 |
| Diff (1-2) |  | -33.3903 | 63.6984 | 31.8492 |  |  |

| group | Method | Mean | 95% CL Mean | | StdDev | 95% CL Std Dev | |
| --- | --- | --- | --- | --- | --- | --- | --- |
| BCA |  | 463.6 | 405.3 | 522.0 | 69.8184 | 46.1621 | 142.1 |
| CON |  | 497.0 | 449.4 | 544.6 | 56.9240 | 37.6367 | 115.9 |
| Diff (1-2) | Pooled | -33.3903 | -101.7 | 34.9194 | 63.6984 | 46.6353 | 100.5 |
| Diff (1-2) | Satterthwaite | -33.3903 | -102.0 | 35.1803 |  |  |  |

| Method | Variances | DF | t Value | Pr > \|t\| |
| --- | --- | --- | --- | --- |
| Pooled | Equal | 14 | -1.05 | 0.3122 |
| Satterthwaite | Unequal | 13.454 | -1.05 | 0.3129 |

| Equality of Variances | | | | |
| --- | --- | --- | --- | --- |
| Method | Num DF | Den DF | F Value | Pr > F |
| Folded F | 7 | 7 | 1.50 | 0.6033 |

**Crypt depth**

**data** d;

input group $ d @@;

datalines;

CON 101.6254

CON 107.45

CON 115.4783

CON 124.062

CON 114.9538

CON 87.599

CON 102.4333

CON 108.144

BCA 97.384

BCA 104.4714

BCA 134.93

BCA 112.362

BCA 98.535

BCA 130.2855

BCA 93.815

BCA 102.1422

;

**proc** **ttest** data=d;

class group;

var d;

**run**;

**Results**

The TTEST Procedure

Variable: d

| group | N | Mean | Std Dev | Std Err | Minimum | Maximum |
| --- | --- | --- | --- | --- | --- | --- |
| BCA | 8 | 109.2 | 15.4866 | 5.4753 | 93.8150 | 134.9 |
| CON | 8 | 107.7 | 11.0180 | 3.8955 | 87.5990 | 124.1 |
| Diff (1-2) |  | 1.5224 | 13.4394 | 6.7197 |  |  |

| group | Method | Mean | 95% CL Mean | | Std Dev | 95% CL Std Dev | |
| --- | --- | --- | --- | --- | --- | --- | --- |
| BCA |  | 109.2 | 96.2935 | 122.2 | 15.4866 | 10.2393 | 31.5195 |
| CON |  | 107.7 | 98.5069 | 116.9 | 11.0180 | 7.2848 | 22.4247 |
| Diff (1-2) | Pooled | 1.5224 | -12.8899 | 15.9347 | 13.4394 | 9.8393 | 21.1952 |
| Diff (1-2) | Satterthwaite | 1.5224 | -13.0366 | 16.0814 |  |  |  |

| Method | Variances | DF | T Value | Pr > \|t\| |
| --- | --- | --- | --- | --- |
| Pooled | Equal | 14 | 0.23 | 0.8240 |
| Satterthwaite | Unequal | 12.641 | 0.23 | 0.8244 |

| Equality of Variances | | | | |
| --- | --- | --- | --- | --- |
| Method | Num DF | Den DF | F Value | Pr > F |
| Folded F | 7 | 7 | 1.98 | 0.3890 |

**Villus height/Crypt depth**

**data** d;

input group $ d @@;

datalines;

CON 4.850907

CON 4.636081

CON 4.913009

CON 3.631672

CON 4.211251

CON 5.748444

CON 4.586754

CON 6.102305

BCA 4.402873

BCA 3.348241

BCA 3.74027

BCA 4.237714

BCA 4.483244

BCA 4.939317

BCA 5.157057

BCA 4.938503

;

**proc** **ttest** data=d;

class group;

var d;

**run**;

**Results**

The TTEST Procedure

Variable: d

| group | N | Mean | Std Dev | Std Err | Minimum | Maximum |
| --- | --- | --- | --- | --- | --- | --- |
| BCA | 8 | 4.4059 | 0.6243 | 0.2207 | 3.3482 | 5.1571 |
| CON | 8 | 4.8351 | 0.7915 | 0.2798 | 3.6317 | 6.1023 |
| Diff (1-2) |  | -0.4292 | 0.7128 | 0.3564 |  |  |

| group | Method | Mean | 95% CL Mean | | Std Dev | 95% CL Std Dev | |
| --- | --- | --- | --- | --- | --- | --- | --- |
| BCA |  | 4.4059 | 3.8840 | 4.9278 | 0.6243 | 0.4128 | 1.2707 |
| CON |  | 4.8351 | 4.1734 | 5.4967 | 0.7915 | 0.5233 | 1.6109 |
| Diff (1-2) | Pooled | -0.4292 | -1.1936 | 0.3353 | 0.7128 | 0.5219 | 1.1242 |
| Diff (1-2) | Satterthwaite | -0.4292 | -1.1975 | 0.3392 |  |  |  |

| Method | Variances | DF | T Value | Pr > \|t\| |
| --- | --- | --- | --- | --- |
| Pooled | Equal | 14 | -1.20 | 0.2485 |
| Satterthwaite | Unequal | 13.28 | -1.20 | 0.2496 |

| Equality of Variances | | | | |
| --- | --- | --- | --- | --- |
| Method | Num DF | Den DF | F Value | Pr > F |
| Folded F | 7 | 7 | 1.61 | 0.5465 |

**T-AOC**

**data** d;

input group $ d @@;

datalines;

CON 0.648

CON 0.646

CON 0.678

CON 0.591

CON 0.672

CON 0.614

CON 0.643

CON 0.644

BCA 0.729

BCA 0.751

BCA 0.676

BCA 0.632

BCA 0.65

BCA 0.585

BCA 0.652

BCA 0.67

;

**proc** **ttest** data=d;

class group;

var d;

**run**;

**Results**

The TTEST Procedure

Variable: d

| group | N | Mean | Std Dev | Std Err | Minimum | Maximum |
| --- | --- | --- | --- | --- | --- | --- |
| BCA | 8 | 0.6681 | 0.0527 | 0.0186 | 0.5850 | 0.7510 |
| CON | 8 | 0.6420 | 0.0284 | 0.0100 | 0.5910 | 0.6780 |
| Diff (1-2) |  | 0.0261 | 0.0423 | 0.0212 |  |  |

| group | Method | Mean | 95% CL Mean | | Std Dev | 95% CL Std Dev | |
| --- | --- | --- | --- | --- | --- | --- | --- |
| BCA |  | 0.6681 | 0.6241 | 0.7122 | 0.0527 | 0.0348 | 0.1073 |
| CON |  | 0.6420 | 0.6183 | 0.6657 | 0.0284 | 0.0188 | 0.0578 |
| Diff (1-2) | Pooled | 0.0261 | -0.0193 | 0.0715 | 0.0423 | 0.0310 | 0.0668 |
| Diff (1-2) | Satterthwaite | 0.0261 | -0.0206 | 0.0728 |  |  |  |

| Method | Variances | DF | T Value | Pr > \|t\| |
| --- | --- | --- | --- | --- |
| Pooled | Equal | 14 | 1.23 | 0.2373 |
| Satterthwaite | Unequal | 10.745 | 1.23 | 0.2433 |

| Equality of Variances | | | | |
| --- | --- | --- | --- | --- |
| Method | Num DF | Den DF | F Value | Pr > F |
| Folded F | 7 | 7 | 3.45 | 0.1246 |

**MDA**

data d;

input group $ d @@;

datalines;

CON 4.231

CON 3.365

CON 3.558

CON 2.885

CON 3.077

CON 3.462

CON 4.712

CON 2.981

BCA 1.442

BCA 3.077

BCA 3.462

BCA 3.173

BCA 4.038

BCA 2.5

BCA 2.308

;

proc ttest data=d;

class group;

var d;

run;

**Results**

The TTEST Procedure

Variable: d

| group | N | Mean | Std Dev | Std Err | Minimum | Maximum |
| --- | --- | --- | --- | --- | --- | --- |
| BCA | 7 | 2.8571 | 0.8505 | 0.3215 | 1.4420 | 4.0380 |
| CON | 8 | 3.5339 | 0.6374 | 0.2253 | 2.8850 | 4.7120 |
| Diff (1-2) |  | -0.6767 | 0.7434 | 0.3847 |  |  |

| group | Method | Mean | 95% CL Mean | | Std Dev | 95% CL Std Dev | |
| --- | --- | --- | --- | --- | --- | --- | --- |
| BCA |  | 2.8571 | 2.0706 | 3.6437 | 0.8505 | 0.5481 | 1.8729 |
| CON |  | 3.5339 | 3.0010 | 4.0667 | 0.6374 | 0.4214 | 1.2972 |
| Diff (1-2) | Pooled | -0.6767 | -1.5079 | 0.1544 | 0.7434 | 0.5389 | 1.1976 |
| Diff (1-2) | Satterthwaite | -0.6767 | -1.5402 | 0.1868 |  |  |  |

| Method | Variances | DF | T Value | Pr > \|t\| |
| --- | --- | --- | --- | --- |
| Pooled | Equal | 13 | -1.76 | 0.1021 |
| Satterthwaite | Unequal | 11.057 | -1.72 | 0.1125 |

| Equality of Variances | | | | |
| --- | --- | --- | --- | --- |
| Method | Num DF | Den DF | F Value | Pr > F |
| Folded F | 6 | 7 | 1.78 | 0.4674 |

**CAT**

**data** d;

input group $ d @@;

datalines;

CON 5.556

CON 2.304

CON 4.743

CON 4.201

CON 7.317

CON 4.065

CON 3.93

CON 2.575

BCA 10.163

BCA 8.943

BCA 8.401

BCA 8.943

BCA 12.195

BCA 10.434

BCA 10.569

BCA 8.808

;

**proc** **ttest** data=d;

class group;

var d;

**run**;

**Results**

The TTEST Procedure

Variable: d

| group | N | Mean | Std Dev | Std Err | Minimum | Maximum |
| --- | --- | --- | --- | --- | --- | --- |
| BCA | 8 | 9.8070 | 1.2689 | 0.4486 | 8.4010 | 12.1950 |
| CON | 8 | 4.3364 | 1.6048 | 0.5674 | 2.3040 | 7.3170 |
| Diff (1-2) |  | 5.4706 | 1.4466 | 0.7233 |  |  |

| group | Method | Mean | 95% CL Mean | | Std Dev | 95% CL Std Dev | |
| --- | --- | --- | --- | --- | --- | --- | --- |
| BCA |  | 9.8070 | 8.7462 | 10.8678 | 1.2689 | 0.8390 | 2.5826 |
| CON |  | 4.3364 | 2.9948 | 5.6780 | 1.6048 | 1.0610 | 3.2661 |
| Diff (1-2) | Pooled | 5.4706 | 3.9193 | 7.0220 | 1.4466 | 1.0591 | 2.2815 |
| Diff (1-2) | Satterthwaite | 5.4706 | 3.9115 | 7.0297 |  |  |  |

| Method | Variances | DF | T Value | Pr > \|t\| |
| --- | --- | --- | --- | --- |
| Pooled | Equal | 14 | 7.56 | <.0001 |
| Satterthwaite | Unequal | 13.293 | 7.56 | <.0001 |

| Equality of Variances | | | | |
| --- | --- | --- | --- | --- |
| Method | Num DF | Den DF | F Value | Pr > F |
| Folded F | 7 | 7 | 1.60 | 0.5505 |

**r-GT**

**data** d;

input group $ d @@;

datalines;

CON 40.9

CON 45.7

CON 59.8

CON 68.5

CON 39.4

CON 43.3

CON 55.1

CON 44.1

BCA 22

BCA 18.1

BCA 20.5

BCA 27.6

BCA 4.7

BCA 55.9

BCA 35.4

BCA 19.7

;

**proc** **ttest** data=d;

class group;

var d;

**run**;

**Results**

The TTEST Procedure

Variable: d

| group | N | Mean | Std Dev | Std Err | Minimum | Maximum |
| --- | --- | --- | --- | --- | --- | --- |
| BCA | 8 | 25.4875 | 15.0469 | 5.3199 | 4.7000 | 55.9000 |
| CON | 8 | 49.6000 | 10.3949 | 3.6752 | 39.4000 | 68.5000 |
| Diff (1-2) |  | -24.1125 | 12.9318 | 6.4659 |  |  |

| group | Method | Mean | 95% CL Mean | | Std Dev | 95% CL Std Dev | |
| --- | --- | --- | --- | --- | --- | --- | --- |
| BCA | 25.4875 | 12.9080 | 38.0670 | 15.0469 | 9.9486 | 30.6246 |  |
| CON | 49.6000 | 40.9096 | 58.2904 | 10.3949 | 6.8728 | 21.1565 |  |
| Diff (1-2) | Pooled | -24.1125 | -37.9805 | -10.2445 | 12.9318 | 9.4677 | 20.3948 |
| Diff (1-2) | Satterthwaite | -24.1125 | -38.1452 | -10.0798 |  |  |  |

| Method | Variances | DF | T Value | Pr > \|t\| |
| --- | --- | --- | --- | --- |
| Pooled | Equal | 14 | -3.73 | 0.0022 |
| Satterthwaite | Unequal | 12.442 | -3.73 | 0.0027 |

| Equality of Variances | | | | |
| --- | --- | --- | --- | --- |
| Method | Num DF | Den DF | F Value | Pr > F |
| FoldedF | 7 | 7 | 2.10 | 0.3502 |

**IL-1β**

**data** d;

input group $ d @@;

datalines;

CON 26.834

CON 28.038

CON 30.414

CON 25.924

CON 26.33

CON 24.905

CON 30.904

CON 26.127

BCA 25.823

BCA 27.136

BCA 29.428

BCA 25.211

BCA 27.036

BCA 30.806

BCA 29.231

BCA 28.436

;

**proc** **ttest** data=d;

class group;

var d;

**run**;

**Results**

The TTEST Procedure

Variable: d

| group | N | Mean | Std Dev | Std Err | Minimum | Maximum |
| --- | --- | --- | --- | --- | --- | --- |
| BCA | 8 | 27.8884 | 1.9166 | 0.6776 | 25.2110 | 30.8060 |
| CON | 8 | 27.4345 | 2.1794 | 0.7705 | 24.9050 | 30.9040 |
| Diff (1-2) |  | 0.4539 | 2.0522 | 1.0261 |  |  |

| group | Method | Mean | 95% CL Mean | | Std Dev | 95% CL Std Dev | |
| --- | --- | --- | --- | --- | --- | --- | --- |
| BCA | 27.8884 | 26.2861 | 29.4907 | 1.9166 | 1.2672 | 3.9007 |  |
| CON | 27.4345 | 25.6125 | 29.2565 | 2.1794 | 1.4410 | 4.4357 |  |
| Diff(1-2) | Pooled | 0.4539 | -1.7469 | 2.6546 | 2.0522 | 1.5025 | 3.2365 |
| Diff(1-2) | Satterthwaite | 0.4539 | -1.7503 | 2.6580 |  |  |  |

| Method | Variances | DF | T Value | Pr > \|t\| |
| --- | --- | --- | --- | --- |
| Pooled | Equal | 14 | 0.44 | 0.6650 |
| Satterthwaite | Unequal | 13.775 | 0.44 | 0.6651 |

| Equality of Variances | | | | |
| --- | --- | --- | --- | --- |
| Method | Num DF | Den DF | F Value | Pr > F |
| Folded F | 7 | 7 | 1.29 | 0.7431 |

**IL-6**

**data** d;

input group $ d @@;

datalines;

CON 54.319

CON 61.323

CON 72.299

CON 69.919

CON 60.976

CON 83.382

CON 84.047

CON 54.672

BCA 57.486

BCA 71.62

BCA 84.712

BCA 63.056

BCA 48.974

BCA 78.37

BCA 70.6

BCA 45.361

;

**proc** **ttest** data=d;

class group;

var d;

**run**;

**Results**

The TTEST Procedure

Variable: d

| group | N | Mean | Std Dev | Std Err | Minimum | Maximum |
| --- | --- | --- | --- | --- | --- | --- |
| BCA | 8 | 65.0224 | 13.8629 | 4.9013 | 45.3610 | 84.7120 |
| CON | 8 | 67.6171 | 11.7948 | 4.1701 | 54.3190 | 84.0470 |
| Diff (1-2) |  | -2.5948 | 12.8705 | 6.4352 |  |  |

| group | Method | Mean | 95% CL Mean | | Std Dev | 95% CL Std Dev | |
| --- | --- | --- | --- | --- | --- | --- | --- |
| BCA |  | 65.0224 | 53.4327 | 76.6121 | 13.8629 | 9.1658 | 28.2148 |
| CON |  | 67.6171 | 57.7564 | 77.4779 | 11.7948 | 7.7984 | 24.0057 |
| Diff (1-2) | Pooled | -2.5948 | -16.3970 | 11.2075 | 12.8705 | 9.4228 | 20.2980 |
| Diff (1-2) | Satterthwaite | -2.5948 | -16.4303 | 11.2408 |  |  |  |

| Method | Variances | DF | T Value | Pr > \|t\| |
| --- | --- | --- | --- | --- |
| Pooled | Equal | 14 | -0.40 | 0.6929 |
| Satterthwaite | Unequal | 13.65 | -0.40 | 0.6930 |

| Equality of Variances | | | | |
| --- | --- | --- | --- | --- |
| Method | Num DF | Den DF | F Value | Pr > F |
| Folded F | 7 | 7 | 1.38 | 0.6806 |

**TNF-α**

**data** d;

input group $ d @@;

datalines;

CON 39.701

CON 40.471

CON 48.944

CON 43.521

CON 50.051

CON 50.97

CON 45.781

BCA 37.762

BCA 39.508

BCA 39.121

BCA 42.193

BCA 43.143

BCA 47.646

BCA 38.734

;

**proc** **ttest** data=d;

class group;

var d;

**run**;

**Results**

The TTEST Procedure

Variable: d

| group | N | Mean | Std Dev | Std Err | Minimum | Maximum |
| --- | --- | --- | --- | --- | --- | --- |
| BCA | 7 | 41.1581 | 3.4493 | 1.3037 | 37.7620 | 47.6460 |
| CON | 7 | 45.6341 | 4.5695 | 1.7271 | 39.7010 | 50.9700 |
| Diff (1-2) |  | -4.4760 | 4.0483 | 2.1639 |  |  |

| group | Method | Mean | 95% CL Mean | | Std Dev | 95% CL Std Dev | |
| --- | --- | --- | --- | --- | --- | --- | --- |
| BCA |  | 41.1581 | 37.9681 | 44.3482 | 3.4493 | 2.2227 | 7.5956 |
| CON |  | 45.6341 | 41.4081 | 49.8602 | 4.5695 | 2.9446 | 10.0624 |
| Diff (1-2) | Pooled | -4.4760 | -9.1908 | 0.2388 | 4.0483 | 2.9030 | 6.6827 |
| Diff (1-2) | Satterthwaite | -4.4760 | -9.2304 | 0.2784 |  |  |  |

| Method | Variances | DF | T Value | Pr > \|t\| |
| --- | --- | --- | --- | --- |
| Pooled | Equal | 12 | -2.07 | 0.0609 |
| Satterthwaite | Unequal | 11.162 | -2.07 | 0.0626 |

| Equality of Variances | | | | |
| --- | --- | --- | --- | --- |
| Method | Num DF | Den DF | F Value | Pr > F |
| Folded F | 6 | 6 | 1.75 | 0.5113 |

**DAO**

**data** d;

input group $ d @@;

datalines;

CON 4.633

CON 3.88

CON 2.953

CON 4.238

CON 5.432

CON 4.6

CON 4.173

CON 4.108

BCA 5.064

BCA 3.016

BCA 2.578

BCA 2.859

BCA 3.174

BCA 3.206

BCA 3.143

BCA 3.365

;

**proc** **ttest** data=d;

class group;

var d;

**run**;

**Results**

The TTEST Procedure

Variable: d

| group | N | Mean | Std Dev | Std Err | Minimum | Maximum |
| --- | --- | --- | --- | --- | --- | --- |
| BCA | 8 | 3.3006 | 0.7523 | 0.2660 | 2.5780 | 5.0640 |
| CON | 8 | 4.2521 | 0.7083 | 0.2504 | 2.9530 | 5.4320 |
| Diff (1-2) |  | -0.9515 | 0.7306 | 0.3653 |  |  |

| group | Method | Mean | 95% CL Mean | | Std Dev | 95% CL Std Dev | |
| --- | --- | --- | --- | --- | --- | --- | --- |
| BCA |  | 3.3006 | 2.6717 | 3.9296 | 0.7523 | 0.4974 | 1.5311 |
| CON |  | 4.2521 | 3.6600 | 4.8443 | 0.7083 | 0.4683 | 1.4415 |
| Diff (1-2) | Pooled | -0.9515 | -1.7350 | -0.1680 | 0.7306 | 0.5349 | 1.1523 |
| Diff (1-2) | Satterthwaite | -0.9515 | -1.7353 | -0.1677 |  |  |  |

| Method | Variances | DF | T Value | Pr > \|t\| |
| --- | --- | --- | --- | --- |
| Pooled | Equal | 14 | -2.60 | 0.0208 |
| Satterthwaite | Unequal | 13.949 | -2.60 | 0.0208 |

| Equality of Variances | | | | |
| --- | --- | --- | --- | --- |
| Method | Num DF | Den DF | F Value | Pr > F |
| Folded F | 7 | 7 | 1.13 | 0.8777 |

**Acetic acid**

**data** d;

input group $ d @@;

datalines;

CON 2287.149

CON 1403.473

CON 1163.455

CON 2130.7

CON 1563.09

CON 2276.061

CON 1382.009

CON 1243.353

BCA 1572.472

BCA 2521.766

BCA 2001.709

BCA 2064.92

BCA 3094.381

BCA 2729.289

BCA 2177.588

BCA 2631.5

;

**proc** **ttest** data=d;

class group;

var d;

**run**;

**Results**

The TTEST Procedure

Variable: d

| group | N | Mean | Std Dev | Std Err | Minimum | Maximum |
| --- | --- | --- | --- | --- | --- | --- |
| BCA | 8 | 2349.2 | 484.6 | 171.3 | 1572.5 | 3094.4 |
| CON | 8 | 1681.2 | 472.6 | 167.1 | 1163.5 | 2287.1 |
| Diff (1-2) |  | 668.0 | 478.6 | 239.3 |  |  |

| group | Method | Mean | 95% CL Mean | | Std Dev | 95% CL Std Dev | |
| --- | --- | --- | --- | --- | --- | --- | --- |
| BCA |  | 2349.2 | 1944.1 | 2754.3 | 484.6 | 320.4 | 986.2 |
| CON |  | 1681.2 | 1286.1 | 2076.3 | 472.6 | 312.5 | 961.9 |
| Diff (1-2) | Pooled | 668.0 | 154.8 | 1181.3 | 478.6 | 350.4 | 754.8 |
| Diff (1-2) | Satterthwaite | 668.0 | 154.7 | 1181.3 |  |  |  |

| Method | Variances | DF | T Value | Pr > \|t\| |
| --- | --- | --- | --- | --- |
| Pooled | Equal | 14 | 2.79 | 0.0144 |
| Satterthwaite | Unequal | 13.991 | 2.79 | 0.0144 |

| Equality of Variances | | | | | |
| --- | --- | --- | --- | --- | --- |
| Method | Num DF | Den DF | F Value | Pr > F |  |
| Folded | F | 7 | 7 | 1.05 | 0.9491 |

**Propionic acid**

**data** d;

input group $ d @@;

datalines;

CON 276.07853

CON 233.8555364

CON 281.3836095

CON 297.336538

CON 214.8658869

CON 272.7334687

CON 222.7412809

CON 213.5195526

BCA 298.2100954

BCA 383.4255816

BCA 249.7888306

BCA 487.4753906

BCA 654.2640818

BCA 372.5044971

BCA 298.7915918

BCA 204.5265994

;

**proc** **ttest** data=d;

class group;

var d;

**run**;

**Results**

The TTEST Procedure

Variable: d

| group | N | Mean | Std Dev | Std Err | Minimum | Maximum |
| --- | --- | --- | --- | --- | --- | --- |
| BCA | 8 | 368.6 | 144.8 | 51.2059 | 204.5 | 654.3 |
| CON | 8 | 251.6 | 33.7468 | 11.9313 | 213.5 | 297.3 |
| Diff (1-2) |  | 117.1 | 105.2 | 52.5776 |  |  |

| group | Method | Mean | 95% CL Mean | | Std Dev | 95% CL Std Dev | |
| --- | --- | --- | --- | --- | --- | --- | --- |
| BCA |  | 368.6 | 247.5 | 489.7 | 144.8 | 95.7593 | 294.8 |
| CON |  | 251.6 | 223.4 | 279.8 | 33.7468 | 22.3125 | 68.6839 |
| Diff (1-2) | Pooled | 117.1 | 4.2914 | 229.8 | 105.2 | 76.9869 | 165.8 |
| Diff (1-2) | Satterthwaite | 117.1 | -4.8469 | 239.0 |  |  |  |

| Method | Variances | DF | T Value | Pr > \|t\| |
| --- | --- | --- | --- | --- |
| Pooled | Equal | 14 | 2.23 | 0.0429 |
| Satterthwaite | Unequal | 7.7579 | 2.23 | 0.0576 |

| Equality of Variances | | | | |
| --- | --- | --- | --- | --- |
| Method | Num DF | Den DF | F Value | Pr > F |
| Folded F | 7 | 7 | 18.42 | 0.0010 |

**Isobutyric acid**

**data** d;

input group $ d @@;

datalines;

CON 75.27401616

CON 85.76935228

CON 71.95069824

CON 85.51874742

CON 79.41246417

CON 76.73206659

CON 81.03011071

CON 81.33610967

BCA 70.5450342

BCA 56.53625982

BCA 77.36032717

BCA 76.90458394

BCA 55.74332841

BCA 55.9085131

BCA 58.70165526

BCA 43.96698591

;

**proc** **ttest** data=d;

class group;

var d;

**run**;

**Results**

The TTEST Procedure

Variable: d

| group | N | Mean | Std Dev | Std Err | Minimum | Maximum |
| --- | --- | --- | --- | --- | --- | --- |
| BCA | 8 | 61.9583 | 11.7914 | 4.1689 | 43.9670 | 77.3603 |
| CON | 8 | 79.6279 | 4.8349 | 1.7094 | 71.9507 | 85.7694 |
| Diff (1-2) |  | -17.6696 | 9.0115 | 4.5057 |  |  |

| group | Method | Mean | 95% CL Mean | | Std Dev | 95% CL Std Dev | |
| --- | --- | --- | --- | --- | --- | --- | --- |
| BCA |  | 61.9583 | 52.1005 | 71.8162 | 11.7914 | 7.7962 | 23.9988 |
| CON |  | 79.6279 | 75.5859 | 83.6700 | 4.8349 | 3.1967 | 9.8403 |
| Diff (1-2) | Pooled | -17.6696 | -27.3335 | -8.0058 | 9.0115 | 6.5975 | 14.2120 |
| Diff (1-2) | Satterthwaite | -17.6696 | -27.8142 | -7.5251 |  |  |  |

| Method | Variances | DF | T Value | Pr > \|t\| |
| --- | --- | --- | --- | --- |
| Pooled | Equal | 14 | -3.92 | 0.0015 |
| Satterthwaite | Unequal | 9.2891 | -3.92 | 0.0033 |

| Equality of Variances | | | | |
| --- | --- | --- | --- | --- |
| Method | Num DF | Den DF | F Value | Pr > F |
| Folded F | 7 | 7 | 5.95 | 0.0314 |

**Butyric acid**

**data** d;

input group $ d @@;

datalines;

CON 945.5054367

CON 280.4274899

CON 239.263399

CON 432.3713967

CON 329.4651699

CON 245.3331429

CON 1156.094097

CON 270.9210154

BCA 721.5851401

BCA 115.7151242

BCA 1430.416064

BCA 368.7218638

BCA 727.9639445

BCA 1255.698463

BCA 779.4694791

BCA 114.083032

;

**proc** **ttest** data=d;

class group;

var d;

**run**;

**Results**

The TTEST Procedure

Variable: d

| group | N | Mean | Std Dev | Std Err | Minimum | Maximum |
| --- | --- | --- | --- | --- | --- | --- |
| BCA | 8 | 689.2 | 484.5 | 171.3 | 114.1 | 1430.4 |
| CON | 8 | 487.4 | 357.5 | 126.4 | 239.3 | 1156.1 |
| Diff (1-2) |  | 201.8 | 425.8 | 212.9 |  |  |

| group | Method | Mean | 95% CL Mean | | Std Dev | 95% CL Std Dev | |
| --- | --- | --- | --- | --- | --- | --- | --- |
| BCA |  | 689.2 | 284.1 | 1094.3 | 484.5 | 320.4 | 986.1 |
| CON |  | 487.4 | 188.5 | 786.3 | 357.5 | 236.4 | 727.7 |
| Diff (1-2) | Pooled | 201.8 | -254.8 | 658.4 | 425.8 | 311.7 | 671.5 |
| Diff (1-2) | Satterthwaite | 201.8 | -258.6 | 662.2 |  |  |  |

| Method | Variances | DF | T Value | Pr > \|t\| |
| --- | --- | --- | --- | --- |
| Pooled | Equal | 14 | 0.95 | 0.3593 |
| Satterthwaite | Unequal | 12.88 | 0.95 | 0.3607 |

Equality of Variances

| Method | Num DF | Den DF | F Value | Pr > F |
| --- | --- | --- | --- | --- |
| Folded F | 7 | 7 | 1.84 | 0.4411 |

**Isovaleric acid**

**data** d;

input group $ d @@;

datalines;

CON 58.2856968

CON 55.0245112

CON 56.8037265

CON 60.9461252

CON 57.1803168

CON 55.4100578

CON 55.9293669

CON 61.2366822

BCA 64.1927312

BCA 51.6008494

BCA 55.2780366

BCA 58.1517532

BCA 93.8355621

BCA 77.284548

BCA 87.4005508

BCA 74.2203509

;

**proc** **ttest** data=d;

class group;

var d;

**run**;

**Results**

The TTEST Procedure

Variable: d

| group | N | Mean | Std Dev | Std Err | Minimum | Maximum |
| --- | --- | --- | --- | --- | --- | --- |
| BCA | 8 | 70.2455 | 15.4489 | 5.4620 | 51.6008 | 93.8356 |
| CON | 8 | 57.6021 | 2.3881 | 0.8443 | 55.0245 | 61.2367 |
| Diff (1-2) |  | 12.6435 | 11.0538 | 5.5269 |  |  |

| group | Method | Mean | 95% CL Mean | | Std Dev | 95% CL Std Dev | |
| --- | --- | --- | --- | --- | --- | --- | --- |
| BCA |  | 70.2455 | 57.3300 | 83.1611 | 15.4489 | 10.2144 | 31.4427 |
| CON |  | 57.6021 | 55.6055 | 59.5986 | 2.3881 | 1.5790 | 4.8605 |
| Diff (1-2) | Pooled | 12.6435 | 0.7895 | 24.4975 | 11.0538 | 8.0928 | 17.4329 |
| Diff (1-2) | Satterthwaite | 12.6435 | -0.3057 | 25.5927 |  |  |  |

| Method | Variances | DF | T Value | Pr > \|t\| |
| --- | --- | --- | --- | --- |
| Pooled | Equal | 14 | 2.29 | 0.0382 |
| Satterthwaite | Unequal | 7.3344 | 2.29 | 0.0543 |

| Equality of Variances | | | | |
| --- | --- | --- | --- | --- |
| Method | Num DF | Den DF | F Value | Pr > F |
| Folded F | 7 | 7 | 41.85 | <.0001 |

**valeric acid**

**data** d;

input group $ d @@;

datalines;

CON 76.9275859

CON 72.406264

CON 70.2946077

CON 72.9162806

CON 70.5325072

CON 71.9856696

CON 84.0636541

CON 79.4025483

BCA 95.3290587

BCA 66.7988858

BCA 85.6856995

BCA 88.4203701

BCA 124.920593

BCA 119.883486

BCA 97.9441144

BCA 66.1080798

;

**proc** **ttest** data=d;

class group;

var d;

**run**;

**Results**

The TTEST Procedure

Variable: d

| group | N | Mean | Std Dev | Std Err | Minimum | Maximum |
| --- | --- | --- | --- | --- | --- | --- |
| BCA | 8 | 93.1363 | 21.5656 | 7.6246 | 66.1081 | 124.9 |
| CON | 8 | 74.8161 | 4.8878 | 1.7281 | 70.2946 | 84.0637 |
| Diff (1-2) |  | 18.3201 | 15.6359 | 7.8180 |  |  |

| group | Method | Mean | 95% CL Mean | | Std Dev | 95% CL Std Dev | |
| --- | --- | --- | --- | --- | --- | --- | --- |
| BCA |  | 93.1363 | 75.1070 | 111.2 | 21.5656 | 14.2586 | 43.8918 |
| CON |  | 74.8161 | 70.7299 | 78.9024 | 4.8878 | 3.2317 | 9.9479 |
| Diff (1-2) | Pooled | 18.3201 | 1.5523 | 35.0880 | 15.6359 | 11.4475 | 24.6594 |
| Diff (1-2) | Satterthwaite | 18.3201 | 0.1763 | 36.4640 |  |  |  |

| Method | Variances | DF | T Value | Pr > \|t\| |
| --- | --- | --- | --- | --- |
| Pooled | Equal | 14 | 2.34 | 0.0344 |
| Satterthwaite | Unequal | 7.7173 | 2.34 | 0.0483 |

| Equality of Variances | | | | |
| --- | --- | --- | --- | --- |
| Method | Num DF | Den DF | F Value | Pr > F |
| Folded F | 7 | 7 | 19.47 | 0.0009 |
